# High throughput whole rumen metagenome profiling using untargeted massively parallel sequencing

**DOI:** 10.1186/1471-2156-13-53

**Published:** 2012-07-02

**Authors:** Elizabeth M Ross, Peter J Moate, Carolyn R Bath, Sophie E Davidson, Tim I Sawbridge, Kathryn M Guthridge, Ben G Cocks, Ben J Hayes

**Affiliations:** 1 Biosciences Research Division, Department of Primary Industries, Bundoora, VIC, 3083, Australia; 2 Dairy Futures Cooperative Research Centre, Bundoora, VIC, 3083, Australia; 3 La Trobe University, Bundoora, VIC, 3086, Australia; 4 Department of Primary Industries, Ellinbank Centre, Ellinbank, VIC, 3820, Australia

**Keywords:** Metagenome profiling, Rumen microbiome, Microbial population comparison

## Abstract

**Background:**

Variation of microorganism communities in the rumen of cattle (*Bos taurus*) is of great interest because of possible links to economically or environmentally important traits, such as feed conversion efficiency or methane emission levels. The resolution of studies investigating this variation may be improved by utilizing untargeted massively parallel sequencing (MPS), that is, sequencing without targeted amplification of genes. The objective of this study was to develop a method which used MPS to generate “rumen metagenome profiles”, and to investigate if these profiles were repeatable among samples taken from the same cow. Given faecal samples are much easier to obtain than rumen fluid samples; we also investigated whether rumen metagenome profiles were predictive of faecal metagenome profiles.

**Results:**

Rather than focusing on individual organisms within the rumen, our method used MPS data to generate quantitative rumen micro-biome profiles, regardless of taxonomic classifications. The method requires a previously assembled reference metagenome. A number of such reference metagenomes were considered, including two rumen derived metagenomes, a human faecal microflora metagenome and a reference metagenome made up of publically available prokaryote sequences. Sequence reads from each test sample were aligned to these references. The “rumen metagenome profile” was generated from the number of the reads that aligned to each contig in the database. We used this method to test the hypothesis that rumen fluid microbial community profiles vary more between cows than within multiple samples from the same cow. Rumen fluid samples were taken from three cows, at three locations within the rumen. DNA from the samples was sequenced on the Illumina GAIIx. When the reads were aligned to a rumen metagenome reference, the rumen metagenome profiles were repeatable (P < 0.00001) by cow regardless of location of sampling rumen fluid. The repeatability was estimated at 9%, albeit with a high standard error, reflecting the small number of animals in the study. Finally, we compared rumen microbial profiles to faecal microbial profiles. Our hypothesis, that there would be a stronger correlation between faeces and rumen fluid from the same cow than between faeces and rumen fluid from different cows, was not supported by our data (with much greater significance of rumen versus faeces effect than animal effect in mixed linear model).

**Conclusions:**

We have presented a simple and high throughput method of metagenome profiling to assess the similarity of whole metagenomes, and illustrated its use on two novel datasets. This method utilises widely used freeware. The method should be useful in the exploration and comparison of metagenomes.

## Background

Ruminants are a group of mammals which include domestic cattle, sheep and goats. The value of domesticated ruminants comes from their ability to convert forages into high quality, high protein products for human consumption through rumen fermentation. The rumen is the first chamber of the ruminant stomach, and it contains symbiotic microorganisms that breakdown ingested food. These microorganisms, which include representatives from all three domains of life: *Eukarya, Bacteria* and *Archaea*[1], provide nutrients, such as volatile fatty acids and bacterial protein to the host animal. Many studies have investigated the symbiotic microorganisms in the rumen because of their link to economically or environmentally important traits such as feed conversion efficiency [2,3], methane production [4,5], and more recently discovery of microbes and enzymes that enable fermentation of biomass for biofuel production [6]. A key challenge here is identifying rumen microbial profiles which are associated, and potentially predictive of these traits. In order to meet this challenge, methods for profiling the rumen microbial population should meet two criteria 1) they should be relatively low cost, so large numbers of individuals can be profiled for testing associations with the above traits, and 2) they should be repeatable, e.g. similar profiles are generated for the same cow, sheep or goat when two repeat samples are taken at the same time.

DNA based methods form the majority of investigations into rumen microbial communities, as culture dependent methods only detect around 11% of species present in the rumen [7]. Polymerase chain reaction (PCR) techniques have allowed the use of culture independent methods to investigate rumen microflora. For example, the 16S genes present in the metagenome (the combined genomes of all organisms present in the sample) are often amplified using universal primers e.g. [7,8]. While PCR based studies that investigate a single, or small selection, of rumen microbial species are informative and necessary, this type of approach may overlook population wide community patterns. Additionally, PCR based methods such as these contain inherent biases [9-11]. Universal primers may not amplify all clades with the same efficiency, thereby biasing abundances towards certain clades. Secondly, universal primers are designed to amplify clades that have been sequenced in the past; resulting in the possibility that novel clades remain undiscovered.

Massive parallel sequencing (MPS) of metagenome DNA without targeted amplification, termed metagenomics, avoids these three issues while enabling a substantial increase in the volume of data produced. In addition, the cost of MPS is falling rapidly. Untargeted MPS involves extracting whole DNA or RNA from the community or tissue of interest. DNA or cDNA is then sequenced, without targeted amplification, using a massively parallel pyrosequencing platform such as the Illumina GAIIx. Recently, untargeted MPS of rumen microbial communities have identified numerous novel gene sequences using deep sequencing of one pooled sample [6]. However, variation between samples of the same type has not yet been shown to be due to within rumen variation (i.e. sampling error) or true biological variation. Here, we develop a method for comparing the variation in rumen meta- genome profiles. The method requires a “reference metagenome”, for example a series of contig sequences from previous sequencing experiments, to which the sample sequence reads are aligned. The “rumen metagenome profile” is then the counts of the reads that align to each contig. For example if there are 200,000 contigs in the database, the profile will be a 200,000 x 1 vector of counts. These profiles can then be analysed using linear mixed models or hierarchical clustering and bootstrap analysis.

One issue with the proposed method is that there is no defined “whole genome” reference for the bovine rumen. This is in contrast to work in human gut metagenomics which has aimed to produce complete whole genome sequences of a subset of common microbial species, with the aim of using these sequences as a reference [12]. It may be possible to use other sequence databases as a reference, such as the prokaryote genomes available from NCBI’s genomes database [13] or the GreenGenes database [14] which contains 16S rRNA sequences. Another possibility is an incomplete rumen derived reference [6], which although not well annotated may be more biologically relevant.

In this study we have sequenced rumen fluid samples from three cows, with repeat sampling, in order to test the hypothesis that rumen metagenome profiles are more similar for repeat samplings of the same cow, than between cows, for cows fed the same diet and sampled at the same time. The method described above was used to generate the rumen metagenome profiles, using a number of different reference sequences. Finally, we recognise that it may be difficult to obtain rumen fluid samples from a very large number of cattle. We also used the method to compare rumen fluid metagenome profiles to faecal metagenome profiles from the same animals, with seven cattle in this experiment, to determine if the metagenome of faeces predicts the rumen fluid metagenome.

## Results

### Sample characteristics

Illumina GAIIx metagenomic sequencing of eleven rumen fluid samples from three lactating dairy cows resulted in over 6 million reads of 146 bp in each library. After trimming based on Phred quality scores, 91.5% of the total basepairs were retained, and 99.08% (91.9 million in total) sequence reads were retained (mean read length after trimming: 135 bp; see Additional file 1 for sample characteristics of each library).

To confirm the sequences were consistent with those expected from micro-organisms in rumen fluid, a traditional taxonomic analysis was performed (Additional file 1) by using BLASTn [15] and BLASTx [16] to assign all reads to a taxon, as well as by extracting 16S-like reads and using BLASTn to assign them to a taxon. The assignments of reads to taxa were indeed generally similar to those observed in other analysis of rumen fluid microflora communities using MPS [17], although samples showed more Bacteriodetes than Firmicutes.

### Generating rumen micro-biome profiles

**Figure 1:**
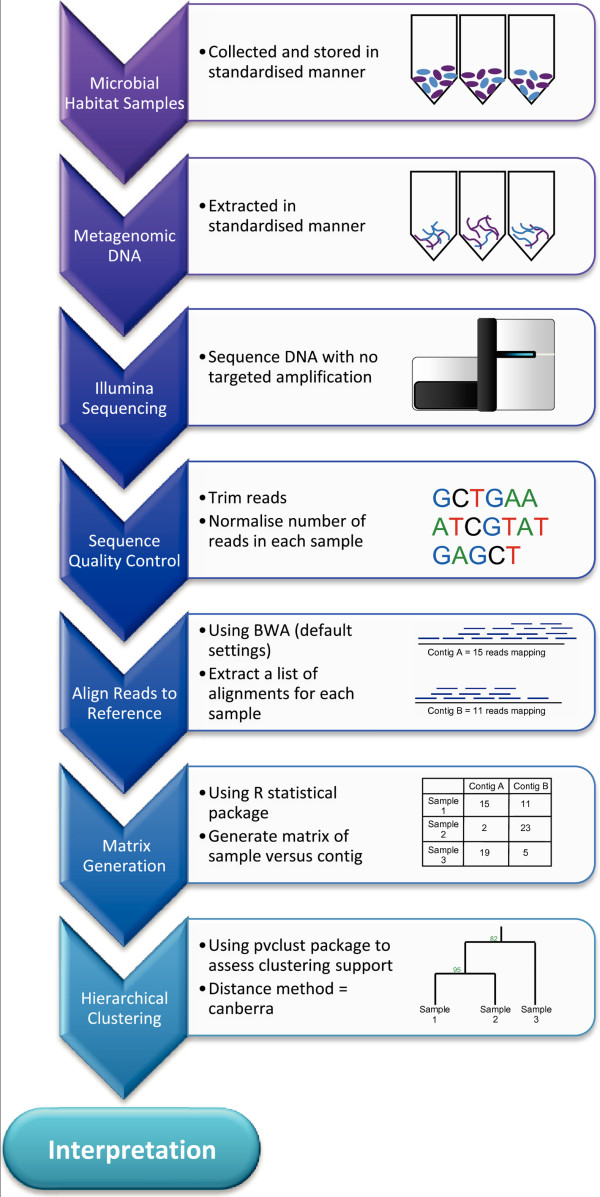
**Flowchart of method.** A representation of the steps involved in the analysis method. Bootstrap support is used to assess the required sequence depth.

A method of comparing the whole microbial community between samples was developed (Figure 1). The method involves aligning the sequence reads to a “reference metagenome”, which consists of either contig sequences or in some cases the whole genome sequence of a number of prokaryotes, using BWA [18]. We define the rumen metagenome profile as the vector of counts of reads aligning to each contig (Figure 1). A number of independent databases were used for this analysis; including both gut derived and non-gut derived references. The non-gut derived references included the 16S GreenGenes database, the NCBI prokaryotes database and a soil metagenome reference (from Minnesota USA) [19]. The gut derived references included two independent rumen MPS contig databases, the first of which was assembled from MPS of bovine and ovine (*Ovis aries*) rumen samples (DPI_rumen), the second was a independently sequenced database (JGI_rumen) which was obtained from Hess et al. [6] and comprised contigs derived from MPS of microbes attached to bovine rumen fibre. The human faeces MPS contig database was used to investigate the effect of a database derived from the gut of a different host species gut.

The similarity between rumen metagenome profiles from the same or different cows can then be visualised following hierarchal clustering. Bootstrap analysis can be used to determine the statistical support for the clustering pattern.

### Reference metagenome effect on clustering pattern

**Figure 2:**
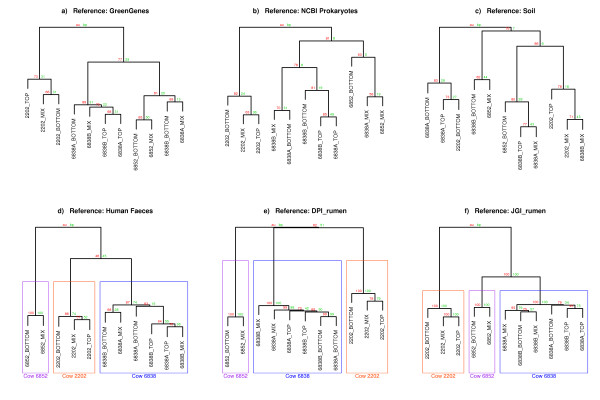
**Hierarchical clustering: between animal variation.** Hierarchical clustering based on alignments of sequence reads to **a)** GreenGenes database, **b)** NCBI Prokaryotes database, **c)** Soil database, **d)** Human Stool database, **e)** DPI_rumen database, **f)** JGI_rumen database. The distance matrix method used was Canberra. Bootstrap (bp) and approximately unbias (au) values were generated using Pvclust [20] with 1000 iterations.

Alignments to the 16S GreenGenes database and the NCBI prokaryotes database were used to generate hierarchical relationships. Further, in order to investigate whether the clustering results for the human faecal reference and rumen derived databases described below were simply an artifact of using a metagenomic database with a large number of contigs from diverse species, we also aligned to a soil metagenome reference. Clustering using both the GreenGenes (Figure 2a) and the NCBI Prokaryotes (Figure 2b) databases distinguished cow 2202 rumen samples from samples taken from the other two cows, while the soil database (Figure 2c) showed no clustering by animal. Clustering using the non-gut references was not strongly supported by the bootstrap or approximately unbias (AU) values.

Sequence reads were aligned to two independent rumen MPS contig databases; DPI_rumen, which was generated from rumen fluid; and JGI_rumen, which was generated from fibre adherent microbes. A human faeces MPS contig database was also used. A total of 5.42% of reads aligned to DPI_rumen, while a total of 0.72% of reads aligned to JGI_rumen. When both rumen databases were combined 6.00% of reads aligned, suggesting that there is little overlap between the two databases, probably due to the difference between rumen fluid and rumen fibre adherent fractions. Less than 1% of reads aligned to the human faeces database, suggesting fairly limited overlap between human stool and rumen fluid microbial populations.

Hierarchical clustering was performed using sequences that aligned to the gut derived references. All three gut based databases were able to separate samples by cow, although in the case of the human faeces database (Figure 2d), the bootstrap support was low. Clustering using DPI_rumen showed separation of libraries by cow, with even distances between animals (Figure 2e). Despite the small proportion of reads aligning, hierarchical clustering using JGI_rumen was also able to separate samples by cow with strong support from bootstrap values (Figure 2f). Unlike DPI_rumen, clustering based on JGI_rumen alignments showed that cows 6852 and 6838 were more similar to each other than they were to cow 2202.

### Required sequence depth for hierarchical clustering

**Figure 3:**
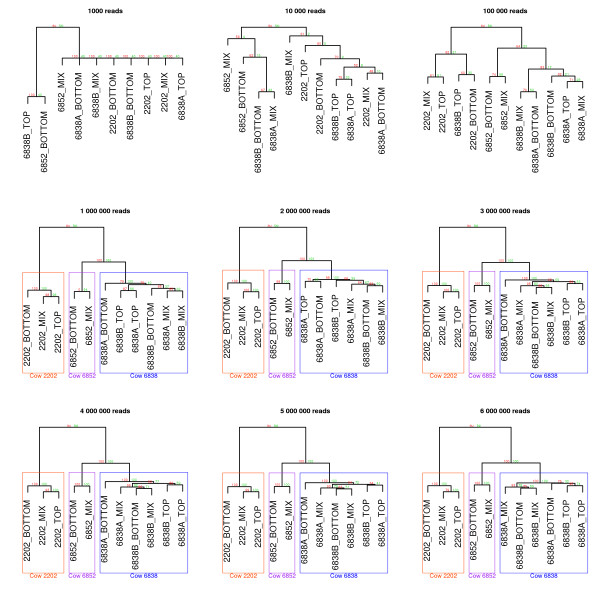
**Hierarchical clustering: sequencing depth effect.** Hierarchical clustering based on alignments of sequence reads to JGI_rumen database, at differing sequencing depths. The distance matrix method used was Canberra. Bootstrap (bp) and approximately unbias (au) values were generated using Pvclust [20] with 1000 iterations.

To assess the amount of sequence data required to successfully obtain separation of rumen fluid samples by cow, sequences from each successful library were sub sampled, and hierarchical clustering performed at different sequencing depths. When the JGI_rumen database was used, less than one million sequence reads resulted in little support for hierarchical structure and clusters contained samples from multiple cows. However once one million reads or more from each sample were utilised, this method was able to separate samples by cow (Figure 3), with very strong support from bootstrap values. Clustering by cow was clearly maintained as the read depth increased up to the maximum of six million paired reads per sample.

### Variation in rumen metagenome profiles explained by cow and rumen fluid sampling location

To assess the amount of variation in the rumen metagenome profiles that was explained by the host animal and the sampling poison, we fitted a linear mixed model (Y_ijk_ = μ + position_i_ + contig_j_ + animal_k_ + e_ijk_) using ASReml [21] to the rumen metagenome profiles generated by aligning reads to the DPI_rumen and JGI_rumen databases (Table 1). Animal, sample position and contig were all fitted as random effects. The reference database had a large effect on the amount of variation explained by the host animal. The effect of the sample position was 18 times smaller than the effect of host animal. Contig was always a large source of variation, highlighting how different rumen microbes have different abundance profiles.

**Table 1:** Linear mixed model analysis of the rumen position experiment

**Source of variation**	**DPI_rumen**	**JGI_rumen**
Host Animal	1.22% ***	9.00% ***
Sampling Position	0.07% ***	0.48% ***
Contig	57.41%	54.03%
Error	41.31%	36.49%

A linear mixed model (Y_ijk_ = μ + position_i_ + contig_j_ + animal_k_ + e_ijk_) was fitted to the read using ASReml [21] count data, where Y = log_n_ transformed counts, position = the position within the rumen that the sample was from, animal = the host animal that samples were taken from. *** p < 0.00001.

### Rumen fluid versus faeces

Collection of rumen samples is more invasive and difficult than collection of faecal samples. For this reason it would be desirable to use faecal samples in place of rumen samples if there is a requirement for sampling from large numbers of animals, and if faecal profiles and rumen metagenome profiles overlapped. We therefore have examined the hypothesis that a cow’s faecal metagenome can be used as a predictor of her rumen metagenome. Rumen fluid and faeces metagenome samples from seven cattle (with both samples taken from all seven cattle) were sequenced on the Illumina HiSeq2000. To the JGI_rumen database, the DPI_rumen database and the human faecal database, 3.97 million sequence read pairs were aligned (Table 2).

**Table 2:** Summary of differences between rumen fluid and faecal samples

	**DPI_rumen**	**JGI_rumen**	**Human faeces**
Percentage aligned (mean ± S.D.)	3.46 ± 2.19	0.68 ± 0.46	0.87 ± 0.40
Contigs unique to faeces	14 635	555	15 090
Contigs unique to rumen fluid	148 861	8 774	6 472
Contigs present in both ^#^	27 313	2 216	3 977
DR contigs (p ≤ 0.01) [FDR]	3 037 [62.8%]	822 [14.0%]	1 168 [21.9%]
DR contigs (p ≤ 0.001) [FDR]	444 [43.0%]	116 [9.9%]	386 [6.6%]
DR^a^ contigs with BLASTn hit	77	88	307

^#^Contigs present in both sample types: faeces and rumen fluid.

DR - significantly differently represented.

FDR - False discovery rate.

^a^ p ≤ 0.001.

**Figure 4:**
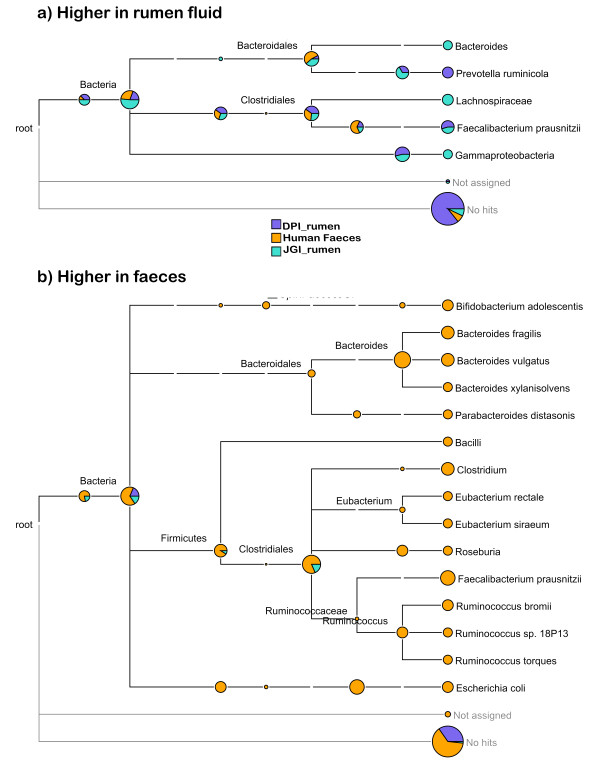
**Differentially represented organisms in rumen fluid and faeces.** The number of sequence read alignments to each contig in three databases were compared between faeces and rumen fluid using a *t*-test. BLASTn was then used to assign contigs that were significantly differently represented (p < 0.001) between rumen fluid and faeces to a taxon. The BLAST output was displayed using MEGAN[22].

When aligning to the two rumen derived databases; unsurprisingly a higher proportion of rumen fluid reads aligned than faecal reads. When aligning to the human faeces database; a higher proportion of faecal reads aligned than rumen fluid. We identified contigs from each of the three databases which were significantly differently represented in faeces compared to rumen fluid (*t*-test corrected for multiple testing; Table 2). False discovery rates for these tests were quite low, between 6.6% and 62.8%. We then used BLASTn to identify the taxa of contigs that were differentially represented between rumen fluid and faeces (p < 0.001; Figure 4). JGI_rumen had the highest percentage of contigs with a successful hit to nt; DPI_rumen had the smallest percentage of contigs with a successful hit to nt. This is likely a reflection of the assembly quality.

**Figure 5:**
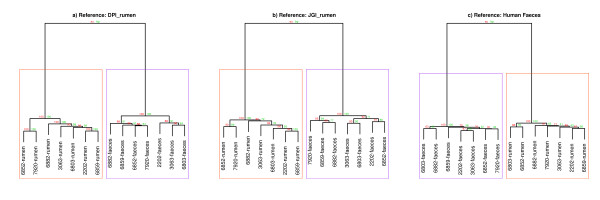
**Hierarchical clustering: comparing faeces to rumen samples.** Hierarchical clustering of rumen and faecal samples from the same animals. Reads were aligned to **a)** DPI_rumen database, **b)** JGI_rumen database, **c)** Human Stool database. The distance matrix method used was Canberra. Bootstrap (bp) and approximately unbias (au) values were generated using Pvclust [20] with 1000 iterations.

When hierarchical clustering was performed on alignments from each sample; all faecal samples clustered together, and all rumen samples clustered together, with very strong bootstrap support for the separation (Figure 5). The deep separation of rumen fluid and faeces samples is in concordance with the large number of unique and differently represented contigs in each sample type (Table 2).

To assess the amount of variation in the metagenome profiles that were explained by the host animal and the sample type (rumen fluid or faeces), we fitted a linear mixed model using ASReml [21] (Y_ijk_ = μ + sample type_i_ + contig_j_ + animal_k_ + e_ijk;_Table 3) to the metagenome profiles that were generated by aligning reads to the DPI_rumen and JGI_rumen databases. Animal, sample type and contig were all fitted as random effects. The effect of sample type was 34 to 49 times greater than the effect of the host animal, reinforcing the large difference in microbial community between the two sample types.

**Table 3:** Linear mixed model analysis of faeces rumen experiment

**Source of variation**	**DPI_rumen**	**JGI_rumen**
Sample Type	28.68% ***	31.56% ***
Host Animal	0.85% ***	0.65% ***
Contig	20.65%	25.70%
Error	49.81%	42.09%

A linear mixed model (Y_ijk_ = μ + sample type_i_ + contig_j_ + animal_k_ + e_ijk_) was fitted to the read using ASReml [21] count data, where Y = log_n_ transformed counts, position = the position within the rumen that the sample was from, animal = the host animal that samples were taken from. *** p < 0.00001.

**Figure 6:**
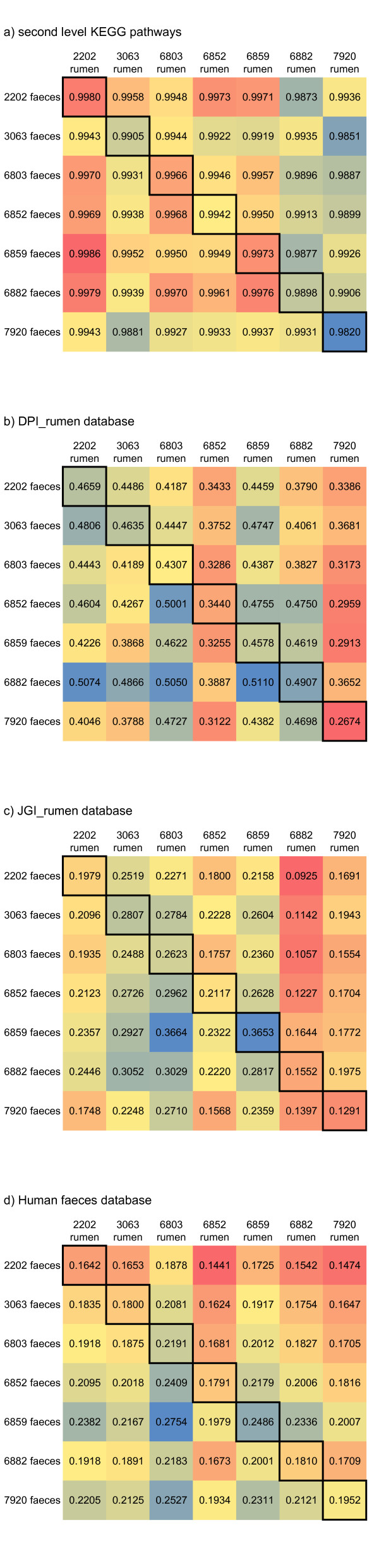
**Heatmap of the correlation between rumen fluid - faeces pairs.** Samples were correlated with each other to asses the degree of similarity between samples. **a)** Correlation in the number of reads assigned to the second level KEGG pathway using BLASTx and MEGAN; **b-d)** correlation in the number of reads assigned to each contig in the database. The results are displayed as a heatmap with the correlation values shown. The correlation for rumen fluid - faeces pairs from the same animal is highlighted.

To test if a cow’s faecal profile was predictive of the rumen fluid metagenome profile, we correlated every rumen metagenome profile with every faecal metagenome profile. We then tested if the correlations were higher for samples which came from the same animal than for between animal samples (Figure 6). The results suggest that faecal - rumen fluid pairs from the same animal did not correlate more strongly with each other than between animals pairs (*t*-test; DPI_rumen p = 0.49; JGI_rumen p = 0.32; Human Faces p = 0.48).

Finally, to examine whether there was a functional correlation between rumen fluid and faeces metagenomes, and to provide an example of a functional analysis that can be performed on this type of data, a basic KEGG analysis was performed. 100 000 reads were aligned to the nr database using BLASTx. The BLAST results were then entered in to MEGAN and the KEGG pathway assignments were extracted. The proportions of second level KEGG assignments were similar between rumen samples and faeces samples. Rumen samples were overall more variable, with standard deviations larger than faeces in 28 out of 36 second level KEGG pathways (paired t- test, p < 0.001). Twelve of the second level KEGG pathways were differently represented between rumen and faeces (paired *t*-test, p < 0.05, false discovery rate = 15%). Translation, Membrane Transport and Cell Motility were all higher in faeces, while Metabolism of Other Amino Acids, Biosynthesis of Other Secondary Metabolites, Signal Transduction, Neurodegenerative Diseases, Nervous System, Cell Communication, Circulatory System, Sensory System and Cardiovascular Diseases were all higher in rumen fluid. The correlation between KEGG assignments were then tested to see if within cow correlations (rumen fluid and faeces from the same cow) were greater than between cow correlations (rumen fluid and faces from different cows). Within cow correlations were not higher than between cow correlations (*t*-test, p = 0.50, Figure 6).

## Discussion

This study demonstrates that untargeted MPS can be used to detect variation between metagenome profiles. The method for deriving rumen microbiome profiles described here, which has similarities to analysing counts of RNA sequence data for example see [23], allows comparison of samples based on the whole population, not just individual species. The method uses data and programs in the public domain. The method is attractive as minimal computational resources are required: less than half a gigabase of sequence data was required to produce repeatable results (clustering of samples within cow) when the bovine rumen was used as an example.

Other methods used to examine the rumen metagenome tend to be either targeted 16S gene studies [24-26], which use some form of amplification and then sequence the amplified products; or they perform whole genomic (shot-gun) untargeted sequencing and then assemble contigs to gain some functional insight into the sample of origin [6,17]. Our method uses the same type of data that is typically generated for functional studies. We have expanded the use of this data to give a profile of species in the metagenome. This type of data can now be used not only for functional analysis, but also to perform an analysis analogous to 16S profiling studies.

No published studies have previously directly addressed the hypothesis that was tested here using MPS: that for the fluid component, within rumen variation is less than between rumen variation. However, it is an assumption implicitly made by studies which take only one technical replicate per animal e.g. [8,17,27]. In this way, our small study appears to confirm previous assumptions made about the rumen fluid microbiome. Samples clustered by host animal when hierarchical clustering was performed, and the linear models confirmed a much larger proportion of variation was due to the host animal than the sample position. The uniformity in the rumen fluid microbiome is likely due to the churning action of the rumen and reticulum [28]. Future sequencing efforts can therefore focus on increasing animal numbers rather than multiple samples per individual.

As rumen fluid samples are difficult to obtain, it would be convenient if there was considerable overlap of the microbe profile between rumen and faecal samples from the same animal. Faecal samples are easy to obtain and hence would lend themselves well to large studies. We were unable to find evidence that rumen fluid and faecal profiles are significantly linked. This was illustrated by the large number of contigs unique to faeces or rumen fluid, as well as identifying many contigs which abundance change significantly from rumen fluid to faeces. Further mixed model analysis showed that the host animal explained little of the variation present; most of the variation was attributed to sample type. We also hypothesised that the microbial profile of a cow’s faeces would correlate more strongly with that cow’s rumen fluid microbial profile than the rumen fluid microbial profile of any other cow. We did not find evidence to support this hypothesis. A more sophisticated analysis may be required to investigate this question in detail.

The differences between rumen and faeces may be a reflection of the function of the two environments - the rumen microbial community may be under strong selection to remain highly functional as the host animal depends on it for digestion, while the faeces may have less restrictions. It is therefore interesting that there was more variation in relative abundance of the functional pathways in rumen than in faeces. Brulc et al. [17] compared rumen sequence to termite. This could be a more relevant comparison as the hindgut of the termite performs a similar function to the rumen - digesting plant material for the host animal.

In our study, a sequence depth of 1 million paired reads was sufficient to detect variation between the rumen fluid micro-biome of different animals. This finding will allow researchers to obtain a relatively inexpensive and detailed summary of the whole rumen fluid metagenome. While not detracting from the importance of deep sequencing, or targeted sequencing of particular genes or species, this study has illustrated a method to obtain a whole rumen fluid metagenome profile, without targeting any particular gene or species group. The production of whole metagenome profiles is likely to be of particular importance for traits where interactions between hundreds or thousands of species may be occurring.

Using multiple databases allowed us to examine the effect of the database source on the clustering pattern. The NCBI prokaryotes reference metagenome and GreenGenes reference metagenome represent well annotated databases. This is the type of database that sequence data is often compared to for taxonomic classification of reads. The soil database reference metagenome is a database of contigs from a different sample type than the test samples. The human faeces reference metagenome represents a database of contigs from a similar sample type as the test samples (both gut samples of mammals). The JGI_rumen database was a database of contigs from a very similar sample type as the test samples (fibre adherent microbes as compared to liquid fraction microbes). The DPI rumen derived database was a databases of contigs from the same sample type as the test samples (although DNA was extracted using a different method, and the sequencing technology used was different). Our samples clustered by animal only when the rumen or human faeces databases were used. The soil database did not produce clustering by cow. This suggests that using a contig database derived from independent sequencing of the same (or similar) community type as the experimental sequences is most appropriate. Interestingly, clustering by host animal was observed even when the database was derived from another species or prepared in a different way to the test samples. Our results suggest that a large proportion of reads aligning to the reference, while desirable, is not absolutely necessary if the reference is biologically relevant to the samples.

Here we have used two databases derived from bovine metagenome sequencing. Other possible sources of bovine metagenome sequence may include Brulc et al. [17] as a rumen derived source or Durso et al. [29] as a faecal source. However, the amount of sequence in each of the above mentioned studies may cause difficulties in assembling a database. We therefore suggest that they be combined with the rumen sequence from this or other [6] studies. A combined bovine metagenome reference assembly could contain reads from multiple sources to take best advantage of sequencing efforts around the world.

In our study we show that the biological relevance of the reference metagenome, in this case rumen derived sequences, is more important than size (as measured by the total number of basepairs in the database). Database size did not have a positive effect on the percentage of sequence reads that aligned. For example the two rumen derived databases showed a five fold difference in the proportion of reads that aligned; despite being approximately equal size (as determined by total summed length of contigs). Hierarchical clustering also appeared unaffected by database size, with the largest (NCBI prokaryotes) and smallest (GreenGenes) databases not clustering samples by cow, but the two intermediate sized rumen derived databases successfully clustering samples by cow. Therefore size is not the most critical feature of the reference metagenome.

The majority of rumen sequences obtained by MPS in this study were novel. It is therefore possible that the reference databases used have limited the power of this study as only a small proportion (0.72%-6.00%) of the sequence information generated was actually used for hierarchical clustering. Despite this, as the contigs present in the rumen derived references represent the most common rumen taxa, it is evident that relative abundance of even the most common rumen species vary enough between animals to allow the microbial profiles of different individuals to be discriminated.

The hierarchical clustering method used in this study should be a useful approach in the analysis of other metagenome datasets. It is similar to methods using RNA sequence to quantify gene expression levels, and it does not require assembly of reads into contigs, or the use of BLAST, both of which require large computational capacities for the volume of data produced by MPS. Also, we have shown that the database need not be from the same environment as the samples, illustrated by the clustering of samples even when databases were prepared in different ways (e.g. DPI_rumen versus JGI_rumen) or from different species (e.g. human faeces). Therefore this method may be particularly useful for novel gut metagenomes, where a reference from closely related species is not available.

The method allows examination of relationships between metagenomes, however the hierarchical clustering can not in itself provide information on which species are driving the hierarchical relationships. One way of dealing with this limitation is to use the metagenome profile matrix to find contigs that are significantly up or down represented between two sample groups, as we did in the comparison of rumen fluid to faeces. Another limitation is that the dendrograms only represent species in the reference database, and hence if key species in the community were missing from the database, the clustering pattern may not represent the community accurately.

This study did not investigate the effect of using the fibre adherent microbes; as such further work may be required to assess if this method is also applicable to the fibre-adherent rumen fraction. Because a database even from a different species (human) could successfully cluster samples by animal, we predict that this method will be applicable to the fibre-adherent rumen fraction, as well as gut metagenomes from other animals.

Another limitation is that the DNA extraction methodology used here may have caused shifts in the proportion of species, or degradation of the DNA. Studies have shown that the method used to extract DNA has a substantial effect on the microbial population observed [30]. However, as all samples were treated in the same manner, these possible effects would have been uniform across all samples. The specific effect of the extraction method we have chosen may be that eukaryotes such as protozoa have been under represented, as the physical disruption used for DNA extraction would likely shear DNA released early in the lysis process. Likewise, the centrifugation of samples before DNA extraction probably caused an under representation of viral sequences, as many viruses would remain in the supernatant. However, neither of these is a concern as it is prokaryote population that we were interested in investigating.

## Conclusion

This study illustrates how untargeted MPS and alignment to a suitable reference metagenome can be used to generate rumen metagenome profiles for individual cattle. These profiles can be used in testing hypotheses such as the one tested here, that there is more variation between rumen metagenome profiles of different cows than of rumen metagenome profiles from repeated samples from the same cow. Our small experiment supported this hypothesis, at least when the samples are taken at the same time and the cows are fed the same diet. The results suggest that when comparing rumen metagenomic profiles, a reference metagenome from a similar habitat is required and that a minimum of one million paired sequence reads is needed. Finally, we found no support for the hypothesis that faecal metagenome profiles and rumen metagenome profiles are more similar when they are from the same cow than when the samples come from different cows, with little correlation between the rumen and faecal metagenomes.

## Methods

This paper presents a simple method for comparing whole microbial populations (Figure 1). First, samples are collected from the microbiome of interest, in this case the rumen and faeces of cattle. Variation in the treatment of samples should be avoided, as this may affect the microbial populations. Next DNA is extracted. The use of the same DNA extraction method on all samples is extremely important, as it has been shown that the chosen DNA extraction method has a large effect on the observed population [30]. The whole DNA extract from each sample is then sequenced on a MPS platform.

To generate the matrix used for hierarchical clustering, quality control is first performed on the sequence reads. Quality control typically involves removing low quality reads, and trimming the ends of reads based on Phred quality scores. The number of reads in each library is then normalised. This removes the effect of library size on the matrix, while retaining information about what does not align to the database. Reads are then aligned to a contig reference. This reference does not have to be well annotated as the method does not rely on identifying specific species or genes. We have found that the database should ideally be from the same type of environment as the samples; although a database generated from a similar environment type (e.g. from a different species, in this case aligning bovine rumen reads to a human faecal reference) can be used if required. This method does not rely on assignments to taxonomy; therefore it can make use of assemblies from whole metagenome sequencing projects as the reference database. Because there is no requirement for 16S genes to be pulled out, the proportion of the metagenome used in the between sample comparison is limited only by the database quality and coverage. The resulting alignments are then used to generate a contig by sample matrix that contains counts of the number of reads from each library that aligned to each contig in the database.

The sample by contig matrix is then used to perform hierarchical clustering. This can be done using freely available software such as the R statistical package [31], specifically the ‘dist’ command to generate a distance matrix. We used the Canberra and binary distance matrix methods. The command ‘hclust’ is then used to generate a dendrogram (we used the method ‘Ward’). To find the support for the generated dendrogram the package pvclust [20] can be used. Low support for the clustering pattern may suggest the need for deeper sequencing of the metagenomes. The dendrogram can be used to interpret the relationships between samples on a whole population level. The distance matrix used in this method could be considered the metagenome equivalent of a genomic relationship matrix, which can be used to infer relationships between individuals based on variation across the genome. Here we infer relationships between samples based on variation across the metagenome.

### Sample collection

Rumen samples were collected from lactating cannulated cows located at the Victorian Department of Primary Industries Ellinbank Centre near Warragul, Victoria Australia (latitude 38 14`S, longitude 145 56`E). A diet of 6.0 kg dry matter of concentrates and *ad libitum* lucerne hay was fed to all cows for two weeks prior to sampling. The concentrate mix contained 4.1 kg dry matter of crushed wheat, 1.5 kg dry matter of cold-pressed canola meal, 0.12 kg dry matter of mineral mix and 0.28 kg dry matter of palabind molasses powder.

For the three cows used in the sample repeatability experiment; after two weeks on the diet, the entire rumen contents were removed through the fistulae, sampled and replaced. The samples referred to as ‘TOP’ were collected from the first third of the rumen contents; these samples generally contained mostly feed material and were quite solid. The samples referred to as ‘BOTTOM’ were collected from approximately the last sixth of rumen contents; these samples were mostly liquid and are possibly representative of a reticulum sample. The samples referred to as ‘MIX’ were collected by mixing together every 20^th^ handful of rumen material; this sample is a representation of the entire rumen. Rumen fluid was squeezed from rumen samples, frozen on dry ice, and then stored at −20°C.

For the seven cows used in the rumen/faeces comparison; rumen fluid was collected as per the MIX sample from the repeatability experiment. Faecal samples were collected on the same morning as rumen samples.

### DNA extraction and sequencing

Thawed rumen fluid was strained through four layers of UV sterilized tulle. 80 mL of filtered rumen fluid was centrifuged at 5000 g for one hour at 4°C. DNA was then extracted from the pellet using PowerMax Soil DNA Isolation Kit (MO-BIO). Faecal samples were prepared in the same manor, omitting the initial centrifugation step.

Library preparation for sequencing was performed using an in-house indexing protocol. The indexes are a short third read of the sequencing run. Briefly, DNA was sheared to 300 bp, adapters were added by ligation, and then indexes were added using PCR. The libraries were then quantified and pooled. Paired-end sequencing of rumen fluid genomic DNA was performed on the GAIIx (sample repeatability experiment) and HiSeq2000 (rumen/faeces comparison experiment) sequencers (Illumina, San Diego, CA). Sequence reads were trimmed so that the average Phred quality score for each read was above 20. If the read length was below 50 after trimming, the read was discarded.

### Hierarchical clustering databases

The DPI_rumen database contained bovine and ovine rumen metagenome sequences that were obtained from whole metagenome sequencing of several samples. Bovine samples were collected from a number of cannulated Holstein-Friesian cows in Victoria, at different times, which were fed ryegrass pasture supplemented with cereal grain. The ovine sample was collected from one sheep, after slaughter at a Victorian abattoir, which had been on a diet of pellets (23.1% crude protein, 4.5% fat). Sequencing of the whole genomic DNA prepared from filtered and unfiltered rumen fluid was performed using the 454 GS-FLX (Roche Diagnostics, Indianapolis, IN), according to manufacturer’s instructions and on 454 FLX Standard (Roche Diagnostics, Indianapolis, IN) and 454 Titanium (Roche Diagnostics, Indianapolis, IN) sequencing platforms at JCVI [32]. See Additional file 1 for a more detailed description.

The SFF files from Roche 454 MPS of the bovine and ovine rumens were assembled into contigs. A combination of Newbler v2.5.3 (release 20101207_1124) and Newbler v2.3 (release 091027_1459) was used. Assemblies were run with default settings, and also stringent parameters: 98% minimum identity, 100 bp minimum length of overlap. The flag ‘-urt’ was used to extend contig length.

The database JGI_rumen consists of contigs previously published by Hess et al. [6]. The GreenGenes database [14] consisted of rRNA sequences. The NCBI prokaryote database [13] contained all sequenced prokaryotes available on the NCBI’s genomes database on the 9^th^ of March 2011. The soil database contained contigs derived from sequencing of top soil [19]. The human faeces database contained contigs from sequencing of human faeces [33]. Additional file 1 contains detailed database characteristics.

### Hierarchical clustering

For the sample repeatability experiment; 6 million reads from each library were analyzed by hierarchical clustering. Sequencing reads were aligned to each of the databases using the default settings of BWA [18]. Alignments were then used to make a sample-by-contig matrix of the number of reads mapping to each contig in the database. Hierarchical clustering was then performed using this matrix in the R statistical package [31]; the package Pvclust [20] was used to compute bootstrap (BP) and approximately unbias (AU) values. For hierarchical clustering two distance matrix methods were used: Binary for presence/absence data and Canberra for counts data.

To test the minimum sequencing depth required for separation of sample by cow, alignments were subsampled by extracting the first 1000, 10 000, 100 000, 1 million, 2 million, 3 million, 4 million, 5 million and 6 million reads from each alignment file. Hierarchical clustering was then performed on the subsamples as described above, using the Canberra distance matrix method.

For the rumen/faeces comparison experiment sequence reads were hierarchically clustered using the same method as the sample repeatability experiment. The R statistical package [31] then used the sample-by-contig matrix to assess the correlations between each faeces sample and each rumen sample. A *t*-test was then performed on these correlation values to assess if the correlation between a cows rumen and faeces profile was higher than the correlation between samples from two different cows.

### Linear mixed models

Statistical analysis was performed using ASReml [21]. The number of reads was modelled using a linear mixed model. For the rumen position experiment, 3 million pairs of reads were aligned to the reference. For the faeces/rumen comparison 4 million pairs of reads were aligned to the reference. A contig by sample matrix (rumen metagenome profile) was generated. Contigs with less than 10 reads aligning were removed from the matrix. The models fitted to the data were Y_ijk_ = μ + sample position_i_ + contig_j_ + animal_k_ + e_ijk_ for the repeated sampling experiment and Y_ijk_ = μ + sample type_i_ + contig_j_ + animal_k_ + e_ijk_ for the rumen faeces comparison. Y was the number of reads mapping, animal was the host animal that the samples were taken from, contig was the contig in the database used, sample type (faeces or rumen fluid) specified the section of the digestive system that the sample was from and sample position (top, bottom or mix) specified the poison within the rumen that the same was taken from. Metagenome profiles generated from alignments to both DPI_rumen and JGI_rumen were used.

### KEGG assignments

For the rumen/faeces comparison experiment 100 000 reads with a minimum Phred quality of 20 for every base, a minimum average quality of 30 across the whole read and a minimum length of 90 basepairs were aligned to nr (last update September 22nd 2011) database on an internal system using BLASTx (minimum e-value 0.2, 5 alignments per read). The resulting output file was then loaded into MEGAN, and the level 2 KEGG assignments were extracted.

### Animal ethics statement

Samples collection was approved by the Victorian Department of Primary Industries (DPI) Agricultural Research & Extension Animal Ethics Committee (Number: AEC 2010_16) and the DPI Metropolitan Animal Ethics Committee (Number: 07/004/DH), and conformed with all relevant regulatory standards.

### Sequence data availability

All raw Illumina sequence data can be obtained freely by contacting the Department of Primary Industries Victoria Biosciences Research Division. The DPI_rumen database has been uploaded to MG-RAST (metagenomics.anl.gov) [ID: 4491686.3].

## Supplementary Material

Additional file 1 **Contains a detailed account of sampling for the DPI_rumen database, a summary of the six databases used including size and number of contigs, a BLAST analysis of the rumen samples, details of the databases used for hierarchical clustering and hierarchical clustering results using a Binary (presence/absence) distance matrix method [**[15,16,22,31]**,**[34]**,**[35]**].** Click here for file
