# Supplementary material for: High throughput whole rumen metagenome profiling using untargeted massively parallel sequencing
Source: BMC Genet. 2012 Jul 2;13:53. doi: 10.1186/1471-2156-13-53 (PMC3464612; doi:10.1186/1471-2156-13-53)
Supplement: Additional file 1 — Contains a detailed account of sampling for the DPI_rumen database, a summary of the six databases used including size and number of contigs, a BLAST analysis of the rumen samples, details of the databases used for hierarchical clustering and hierarchical clustering results using a Binary (presence/absence) distance matrix method [[15,16,22,31],[34],[35]]. [file 1471-2156-13-53-S1.pdf]

Summary

Variation in composition of microorganisms in the rumen of cattle (*Bos taurus*), and the role of the host in controlling this variation, is of great interest because of possible links to feed conversion efficiency and methane emission levels. The resolution of studies investigating rumen microbial communities may be improved by utilizing untargeted massively parallel sequencing (MPS), that is, sequencing without targeted amplification of genes, as many rumen microorganisms resist culture. The ability of MPS to detect between animal variation in the microbial community of rumen fluid is unknown. Rather than focussing on individual organisms within the rumen, our aim was to derive a method for using MPS data to generate quantitative rumen micro-biome profiles. We then investigate whether these profiles are repeatable for individual cows, and validate the method by comparing rumen metagenomes to faecal metagenomes from the same animals.

Rumen fluid was extracted from three cows, with repeated sampling for each cow as well as sampling from different locations within the rumen. Using Illumina sequencing of rumen fluid extract, followed by alignment of sequence to a number of gut references, we demonstrated that there is detectable variation between rumen fluid microflora profiles of individual cattle. There was less variation within samples from the same cow, regardless of the position of sampling of fluid within the rumen, than between cows. This analysis was enhanced by the availability of several independent, biologically relevant metagenome references, including two derived independently

from rumen samples. We further show that this method only requires three million paired sequence reads per sample to detect biological variation in the rumen fluid microbial communities. Interestingly, we also show that gut metagenome reference databases from other species, in this case human, produce a similar clustering pattern, albeit with much lower statistical support. Finally we validate this method by comparing rumen metagenomes to faecal metagenomes from the same animals.

This study shows how short read MPS can be used to detect population wide rumen metagenome differences between individual cattle, and that the observed differences are due to true biological variation, not sampling error. These results represent a proof of principle that short read MPS data can be used to profile rumen fluid metagenomes, this will allow future studies into traits likely to be influenced by the microbial population then the rumen (such as enhanced feed conversion efficiency or decreased methane production). The finding that distinct rumen profiles can be identified with only three million paired sequence reads opens the possibility of large scale studies to detect associations between rumen microbiome profiles and feed conversion efficiency and methane emission levels.

Detailed description of DPI_rumen sequence database

The database is made up of bovine and ovine rumen sequence, obtained from several samples. Bovine samples were collected from a number of cannulated Holstein-Friesian cows in Victoria, at different times, which were fed ryegrass pasture supplemented with cereal grain. The ovine sample was collected from one sheep, after slaughter at a Victorian abattoir, which had been on a diet of pellets (23.1% crude protein and 4.5% fat of dry matter). The filtered bovine DNA samples came from cows on a diet of 4 kg lucerne hay, 7 kg pasture silage, and 3 kg cracked wheat (estimated on group basis, dry matter), and had last eaten 4 hr prior to sampling. Generally, approximately 1 L of rumen fluid was collected from the animal and passed through a 1 mm sieve to remove plant material before being kept on ice to return to the lab, where different samples were treated in different ways.

On one occasion, the rumen of one cow was fractionated whereby the sample was centrifuged (1000 g, 10 mins) to create two fractions – the supernatant and the pellet, both of which were put through a series of descending size filters. The last filters, 0.1 µm and 0.8 µm, were collected and frozen until needed. Genomic DNA was extracted from the bacterial these filters by traditional DNA methods which included subjecting the filters to enzymatic treatment, SDS lysis and freeze-thaw cycles, which were then cleaned up with proteinase K and phenol:chloroform extraction, before ethanol precipitation, and a further clean up procedure using a CTAB extraction to remove carbohydrates. Sequencing of the genomic DNA prepared from the 0.1 µm and 0.8 µm filters was undertaken by pyrosequencing in-house using the 454 GS-FLX (1.5 plates in all) with the appropriate Roche kit, according to manufacturer's instructions.

On a different occasion, rumen fluid from two cows were combined. A sample was centrifuged (3500rpm, 10 min) and the supernatant removed. The pellet was resuspended and washed in extraction buffer (1 x TE buffer, 50mM EGTA pH 8, 50mM EDTA pH 8), before being pelleted and resuspended in a further volume of the buffer. Genomic DNA was extracted using a combination of the lysis steps above and steps from the Promega Wizard Genomic kit to digest RNA and remove proteins, before cleaning up with phenol and phenol/chloroform/IAA extractions, and traditional isopropanol DNA precipiation. Extracted DNA was agarose gel purified, and cleaned up with β-agarase (New England Biolabs), according to manufacturer's instructions. Ovine DNA was extracted in the same way from a rumen sample taken from a single animal.

Sequencing of these "unfractionated" rumen DNA samples was done at JCVI on 454 FLX and 454 Titanium machines. A plate of bovine rumen DNA was run on each of these machines, while one plate of ovine rumen DNA was sequenced on the 454 Titanium.

Table S1. Details of each library including size, read length, trimming information and percent of reads that align to each database.

	Before Quality Control^			After Quality Control^					Alignments of database~						
Sample	Size	Number of reads	Mean Read Length	Size	Number of reads	Mean Read Length	% Kept reads	% Kept size	GreenGenes	NCBI Prokaryotes	DPI_Rumen	JGI_Rumen	Combined Rumen	Soil	Human Faeces
2202_BOTTOM	886,391,112	6,071,172	146	812,374,926	6,016,704	135.02	99.10%	91.65%	0.074%	1.181%	5.190%	0.913%	5.957%	0.0072%	0.501%
2202_MIX	1,111,274,328	7,611,468	146	1,011,066,397	7,536,884	134.15	99.02%	90.98%	0.056%	0.768%	4.356%	1.066%	5.294%	0.0078%	0.485%
2202_TOP	1,099,982,396	7,534,126	146	1,003,947,655	7,464,802	134.49	99.08%	91.27%	0.062%	0.931%	4.954%	1.040%	5.844%	0.0067%	0.491%
6838A_BOTTOM	1,212,569,128	8,305,268	146	1,118,727,448	8,243,610	135.71	99.26%	92.26%	0.073%	0.605%	5.334%	0.548%	5.742%	0.0069%	0.541%
6838A_MIX	1,159,801,516	7,943,846	146	1,055,018,613	7,869,604	134.06	99.07%	90.97%	0.079%	0.602%	5.914%	0.678%	6.441%	0.0074%	0.584%
6838A_TOP	1,293,562,044	8,860,014	146	1,191,583,028	8,788,851	135.58	99.20%	92.12%	0.069%	0.551%	5.015%	0.576%	5.461%	0.0074%	0.469%
6838B_BOTTOM	960,547,140	6,579,090	146	875,261,340	6,521,715	134.21	99.13%	91.12%	0.080%	0.622%	5.803%	0.635%	6.286%	0.0089%	0.562%
6838B_MIX	1,031,436,856	7,064,636	146	949,626,825	7,008,321	135.5	99.20%	92.07%	0.066%	0.466%	4.852%	0.614%	5.341%	0.0070%	0.493%
6838B_TOP	1,360,017,740	9,315,190	146	1,256,757,714	9,246,067	135.92	99.26%	92.41%	0.069%	0.551%	5.108%	0.594%	5.571%	0.0068%	0.505%
6852_BOTTOM	1,285,146,312	8,802,372	146	1,169,985,433	8,715,394	134.24	99.01%	91.04%	0.077%	0.660%	7.202%	0.645%	7.660%	0.0067%	0.601%
6852_MIX	951,806,996	6,519,226	146	872,256,834	6,461,951	134.98	99.12%	91.64%	0.071%	0.577%	5.902%	0.653%	6.411%	0.0064%	0.544%
6852_TOP	372,884	2,554	146	340,638	2,525	134.91	98.86%	91.35%	-	-	-	-	-	-	-
Unknown	1,194,439,724	8,181,094	146	1,080,435,222	8,060,345	134.04	99.10%	90.46%	-	-	-	-	-	-	-
Total/Mean*	13,547,348,176	92,790,056	146	12,397,382,073	91,936,773	134.85	99.08%	91.51%	0.071%	0.683%	5.421%	0.724%	6.001%	0.0072%	0.5251%

*Size and Number of Reads are given as total, all other collums are shown as means.

^Quality Control referes to trimming of reads based on Phred quality score.

~Alignments were not performed with reads that were not assigned to a samples, nor from the failed library (6852 TOP).

Table S2. Characteristics of databases used in hierarchical clustering analysis.

Database	Total Size	Number of Contigs	Source	Description
GreenGenes	163 MB	108,453	http://greengenes.lbl.gov/	16S rRNA gene sequence alignment database downloaded March 25th 2011.
NCBI Prokaryotes	4,723 MB	2,390	ftp://ftp.ncbi.nih.gov/genomes/Bacteria/	NCBI's sequenced prokaryotes, downloaded 9th of March 2011.
soil	154 MB	139,340	Tringe et al. [1]	Contigs from sequencing of clay loam surface soil from Waseca County, Minnesota.
human_stool	4, 010 MB	3,415,004	http://www.hmpdacc.org/HMASM/	Contigs from the sequencing of human faeces. Only the first 4GB was used.
DPI_rumen	480 MB	1,124,380	This Study	Rumen derived database of assembled reads from Roche 454 sequencing of bovine (*Bos taurus*) and ovine (*Ovis aries)* rumen fluid samples. Animals were located in Victoria Australia.
JGI_rumen	545 MB	26,042	Hess et al. [2]	Rumen derived database of assembled reads from Illumina sequencing of bovine rumen samples.

Taxonomic Analysis

To give a traditional overview of the rumen samples used to test the method, BLAST was used. BLAST was performed on an internal system, with a minimum E-value of 0.02. The databases used for the BLASTn algorithm [1] were nt (from NCBI; last updated January 2011) and bacterial genomes (from NCBI; last updated March 2011). The databases used for the BLASTx algorithm [2] were nr (from NCBI; last updated March 2011) and bacterial peptides (from NCBI; last updated February 2011). Reads with homology to 16S rRNA genes were extracted from the dataset using PHYLOSHOP [3]. BLAST results were analyzed with MEGAN [4-5] using the default minimum homology cutoffs (genus 95%, family 90%, order 85%, class 80%, phylum 75%), and R [6].

Sequence reads were compared to publicly available eukaryote and prokaryote sequences using BLASTn [1] and BLASTx [2] to obtain a more traditional profile of the species present in each sample. Only 2.1% of reads produced a significant alignment using the nucleotide (nt) database (BLASTn), but when the non-redundant protein sequences (nr) database (BLASTx) was used, 49.2% produced a significant alignment, with an average identity of 72.8%. When a microbial genomes database was used (BLASTn), 3.4% of reads produced significant alignments, while the microbial proteins database (BLASTx) identified 44.5% of sequences with an average identity of 70.7%. Reads with homology to 16S rRNA genes were then extracted from the dataset using PHYLOSHOP [3], BLASTn was used to assign the reads to taxa. BLAST results were then analyzed using MEGAN v4.40.5 [4-5].

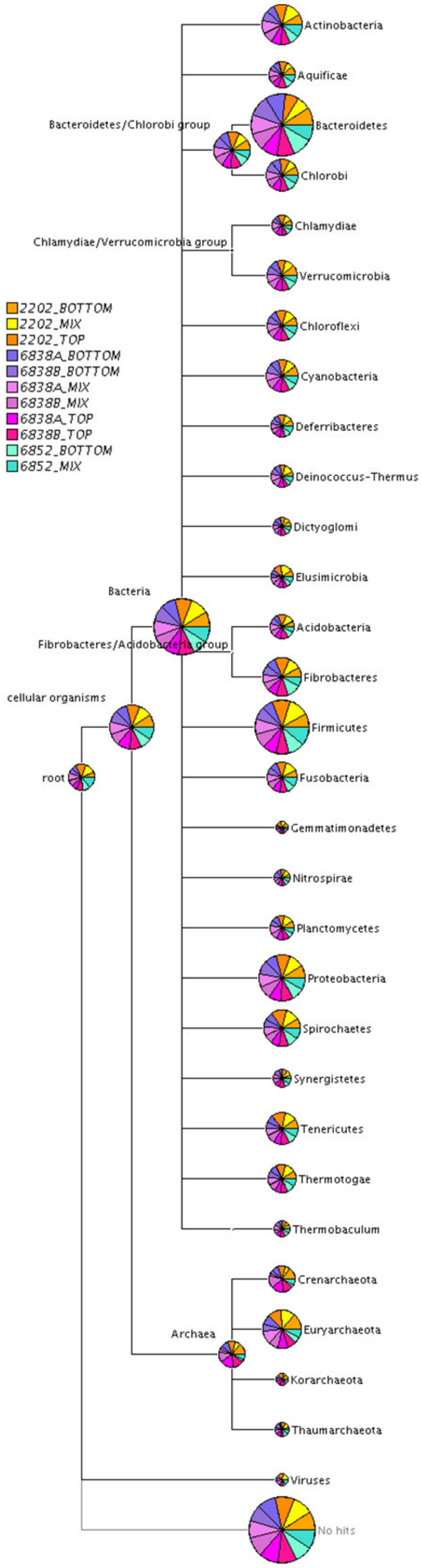

Figure S1 - BLASTx phyla results using all reads

Phyla present in each sequence library as determined by BLASTx with a bacterial proteins database. MEGAN v4.40.5 was used to graphically represent the data. The size of each piegraph represents the proportion of reads in those taxa. Piegraphs at higher taxa nodes represent reads which did not meet the conserved identity for lower taxa classification. The relative size of each slice of piegraph represents the relative proportion of reads from each library in each taxon.

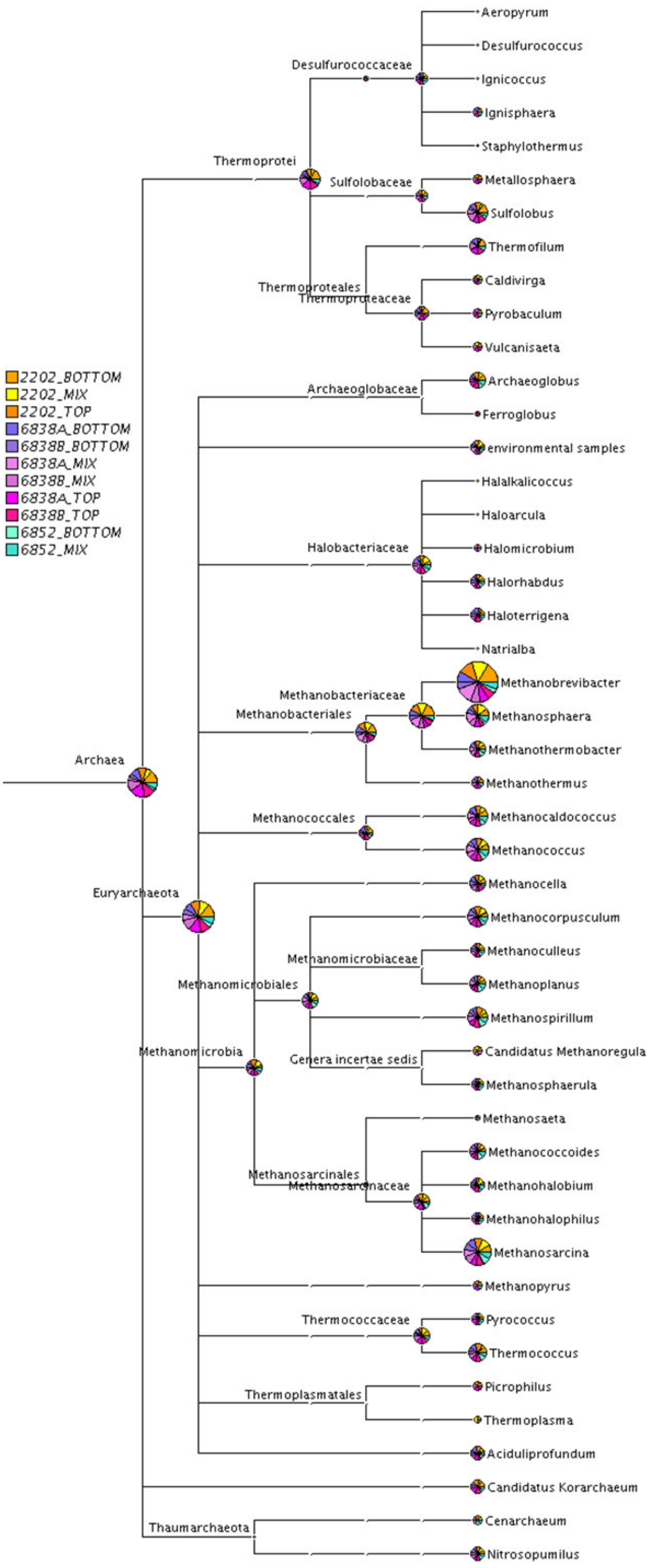

Figure S2 - BLASTx Archaeal genera results using all reads

Archaeal genera present in each sequence library as determined by BLASTx with a bacterial proteins database. MEGAN v4.40.5 was used to graphically represent the data. The size of each piegraph represents the proportion of reads in those taxa. Piegraphs at higher taxa nodes are reads which did not meet the conserved identity for lower taxa classification. The relative size of each slice of piegraph represents the relative proportion of reads from each library in each taxon. The percentage of reads assigned to each taxon can be found in Table S2.

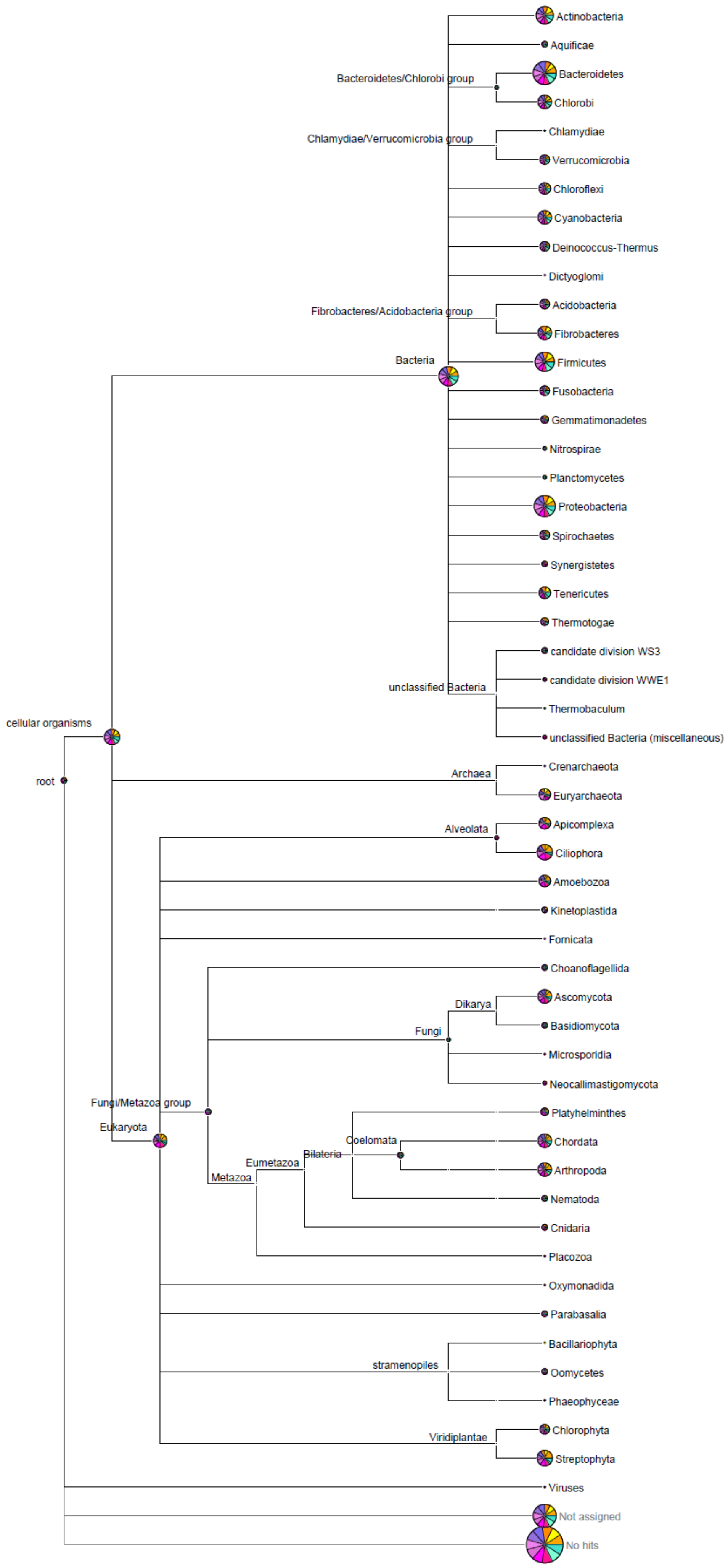

Figure S3 - BLASTn phyla results using all reads

Genera present in each sequence library as determined by BLASTn with nt database. MEGAN v4.40.5 was used to graphically represent the data. The size of each piegraph represents the proportion of reads in those taxa. Piegraphs at higher taxa nodes represent reads which did not meet the conserved identity for lower taxa classification. The relative size of each slice of piegraph represents the relative proportion of reads from each library in each taxon.

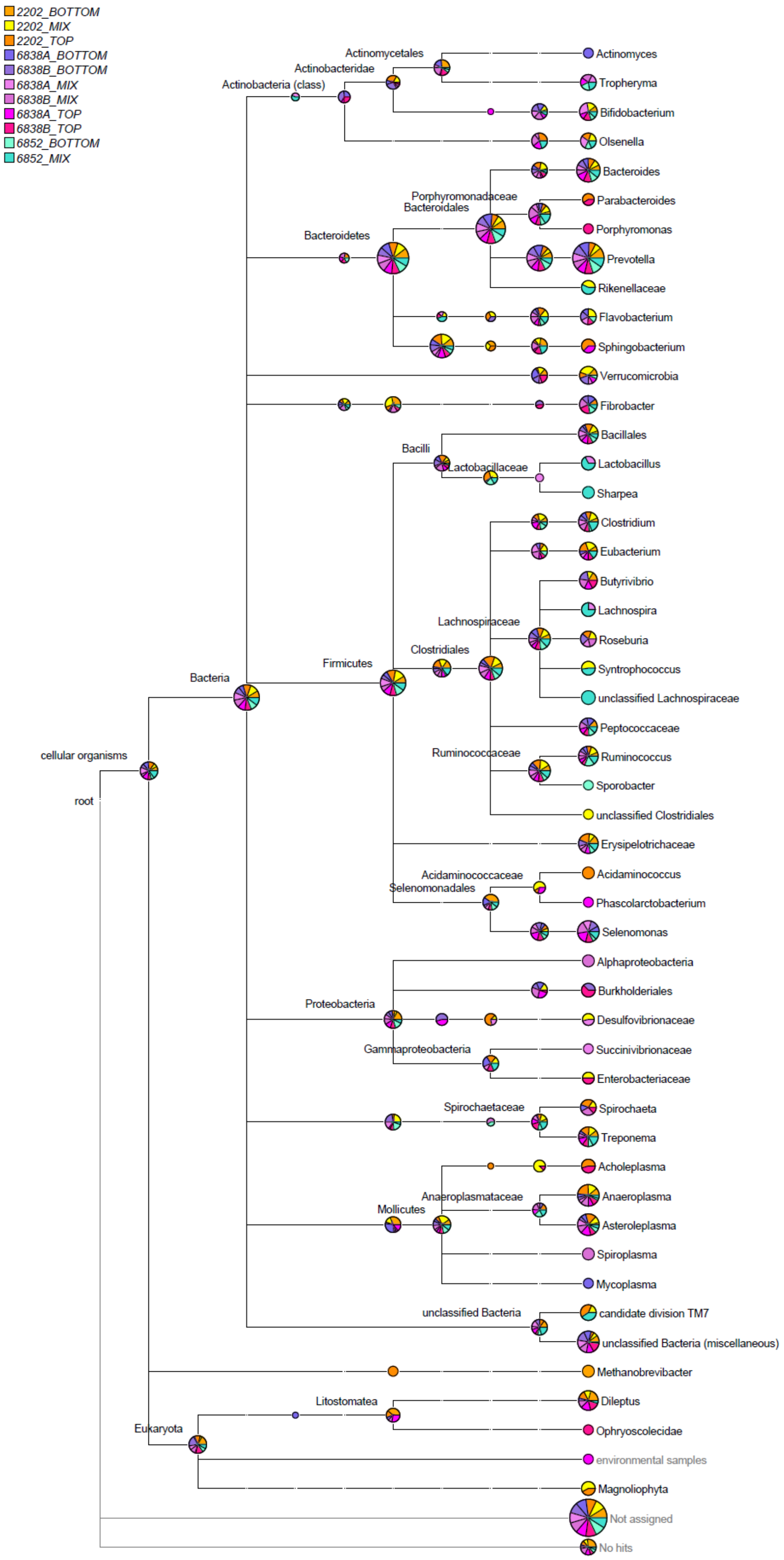

Figure S4 - BLASTn taxa results using 16S assigned reads

Genera present in reads with homology to 16S rRNA genes (extracted from the dataset using PHYLOSHOP [3]) as determined by BLASTn with nt database. MEGAN v4.40.5 was used to graphically represent the data. The size of each piegraph represents the proportion of reads in those taxa. Piegraphs at higher taxa nodes represent reads which did not meet the conserved identity for lower taxa classification. The relative size of each slice of piegraph represents the relative proportion of reads from each library in each taxon.

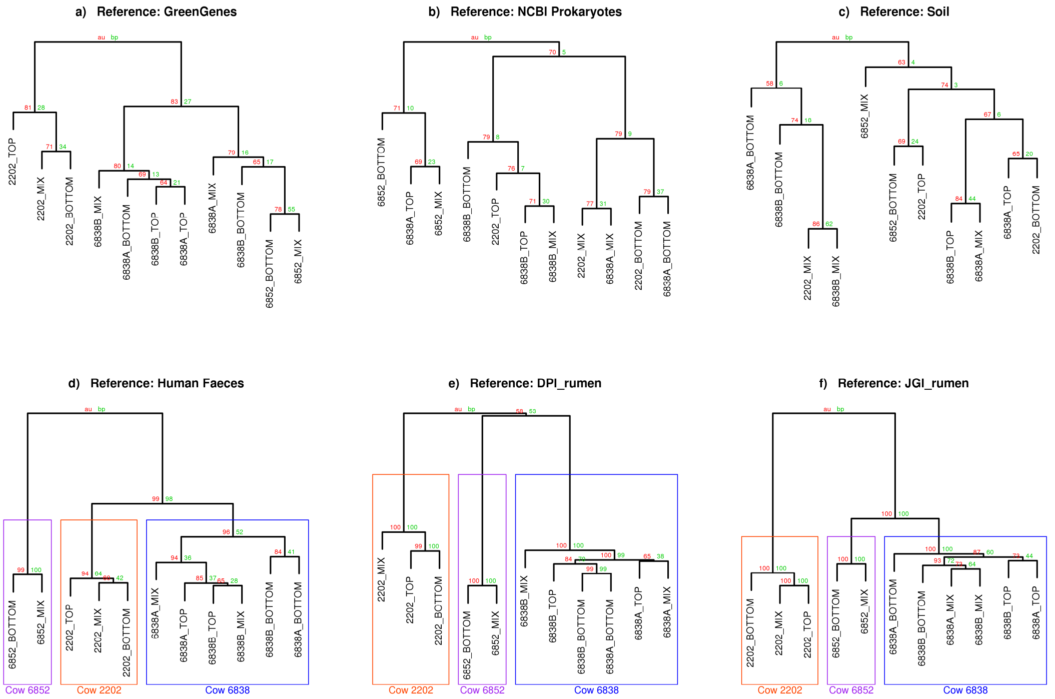

Figure S5 - Hierarchical clustering: Binary between animal variation

Hierarchical clustering based on alignments of sequence reads to a) GreenGenes database, b) NCBI Prokaryotes database, c) Soil database, d) Human Stool database, e) DPI_rumen database, f) JGI_rumen database. The distance matrix method used was Binary. Bootstrap (bp) and approximately unbias (au) values were generated using Pvclust [29] with 1000 iterations

1. Zhang Z, Schwartz S, Wagner L, Miller W: **A Greedy Algorithm for Aligning DNA Sequences.** *J Comput Biol* 2000, **7:**203-214.
2. Altschul SF, Madden TL, Schäffer AA, Zhang J, Zhang Z, Miller W, Lipman DJ: **Gapped BLAST and PSI-BLAST: a new generation of protein database search programs.** *Nucleic Acids Res* 1997, **25:**3389-3402.
3. Shah N, Tang H, Doak TG, Ye Y: **Comparing bacterial communities inferred from 16S rRNA gene sequencing and shotgun metagenomics.** *Pac Symp Biocomput* 2011**:**165-176.
4. Huson DH, Auch AF, Qi J, Schuster SC: **MEGAN analysis of metagenomic data.** *Genome Res* 2007, **17:**377-386.
5. Huson DH, Mitra S, Ruscheweyh H-J, Weber N, Schuster SC: **Integrative analysis of environmental sequences using MEGAN4.** *Genome Res* 2011**:**doi: 10.1101.
6. R Development Core Team: **R: A Language and Environment for Statistical Computing.** Vienna, Austria: R Foundation for Statistical Computing; 2011.
